# Real-World Outcomes of Biologic Therapy for Severe Asthma in a High-Burden Urban Population

**DOI:** 10.7759/cureus.88756

**Published:** 2025-07-25

**Authors:** Justin Louie, Anandu M Anto, Sindhaghatta Venkatram, Charnicia Huggins, Gilda Diaz-Fuentes

**Affiliations:** 1 Pharmacy, BronxCare Health System, Bronx, USA; 2 Internal Medicine, BronxCare Health System, Bronx, USA; 3 Pulmonary and Critical Care, BronxCare Health System, Bronx, USA

**Keywords:** asthma management, biologic therapy, inner-city hospital, real-world evidence, severe asthma

## Abstract

Background

Asthma prevalence and mortality are disproportionately higher in underserved areas such as the Bronx compared to other regions of New York City. Biologic therapy is recommended for severe asthma but may present challenges in access and adherence among patients in low-resource settings. This study evaluates the real-world effectiveness and feasibility of biologic therapy in an inner-city cohort.

Methods

We conducted a retrospective cohort study of adult patients with severe asthma treated at an urban outpatient pulmonary and allergy clinic. Patients who received biologic therapy for at least six months between January 2020 and August 2023 were included. Outcome measures included Asthma Control Test (ACT) scores, asthma exacerbation rates, oral corticosteroid (OCS) use, lung function (forced expiratory volume in one second (FEV_1_) and forced vital capacity (FVC)), and emergency department (ED) visits. Adherence was assessed using the proportion of days covered (PDC).

Results

Out of the 110 patients receiving biologics in our clinic, 44 met the inclusion criteria. The mean age was 63 years; 93% of patients self-reported Hispanic or African American race, and 86% were insured through Medicaid. Benralizumab was the most commonly prescribed biologic, as most of our patients had severe eosinophilic asthma. Following biologic therapy, ACT scores improved significantly (from 15 ± 4.48 to 20 ± 3.8, p < 0.001), ED visits declined (from 54 to 23, p = 0.004), and median daily systemic steroid dosage decreased (from 11.25 mg to 7.5 mg, p < 0.001). Mean FEV_1_ increased from 73.64% to 78% predicted (p = 0.059). Among patients on biologics for over one year, the average PDC was 78%.

Conclusions

Biologic therapy appears to be both feasible and effective for treating severe asthma in underserved urban populations. Through a structured multidisciplinary care model, patients in real-world inner-city settings can achieve improved asthma control, reduced healthcare utilization, and strong treatment adherence as evidenced by a PDC of 78%. These findings highlight the importance of equitable access to advanced therapies in populations historically underrepresented in clinical trials.

## Introduction

Asthma is a chronic non-communicable disease that typically begins in childhood or adolescence, although it can also develop in adulthood [1]. It is characterized by persistent airway inflammation and bronchoconstriction, which negatively affect work, education, and daily activities. Common symptoms include wheezing, shortness of breath, chest tightness, and coughing, which vary in intensity and frequency [2]. Globally, asthma affects an estimated 262 million people and is responsible for approximately 455,000 deaths annually [3].

Allergic asthma, the most common phenotype, is characterized by sensitization to environmental triggers, with symptoms occurring upon exposure [4]. Asthma severity is classified as intermittent, mild, moderate, or severe. In some patients with severe asthma, the disease remains uncontrolled despite adherence to maximally tolerated, optimized high-dose inhaled corticosteroids (ICS) and long-acting beta-agonists (LABA). Approximately 3.7% of individuals with asthma fall into the severe category [5]. These patients experience higher rates of hospitalization, increased side effects from oral corticosteroids (OCS), and poorer quality of life (QOL) compared to those with well-controlled disease [6].

The Global Initiative for Asthma (GINA) guidelines recommend the use of long-acting muscarinic antagonists (LAMA) and/or biologic therapy as a last-line treatment, particularly for patients with type 2 inflammation. Biologic therapies target type 2 inflammatory mediators, such as immunoglobulin E (IgE), interleukin-4 (IL-4) and interleukin-5 (IL-5), and thymic stromal lymphopoietin (TSLP), thereby reducing asthma exacerbations and OCS use while improving QOL and lung function [7,8].

Current Food and Drug Administration (FDA)-approved biologics for asthma include omalizumab (Xolair®), mepolizumab (Nucala®), dupilumab (Dupixent®), benralizumab (Fasenra®), and tezepelumab-ekko (Tezspire®), with omalizumab being the first to be approved in 2003. However, the clinical trial data supporting the FDA approval of these biologics primarily involved populations with limited racial diversity. The proportion of White participants in these trials ranged from 75% for omalizumab, 78%-95% for mepolizumab, 78%-94% for dupilumab, 73%-93% for benralizumab, and 62%-92% for tezepelumab-ekko [9-12]. This lack of representation is concerning because racial and ethnic minority groups experience higher asthma prevalence and greater disease severity, including increased rates of emergency department (ED) visits, hospitalizations, and mortality [13,14]. The underrepresentation of these populations in pivotal trials limits our understanding of how biologic therapies perform in those most affected by asthma. In addition, studies of biologic use in minority populations treated for conditions other than asthma have found both lower adherence to biologic therapy and earlier discontinuation [15,16]. As the use of biologics continues to expand, evaluating their real-world effectiveness across diverse racial, ethnic, and socioeconomic groups becomes essential.

The Bronx remains the New York City borough most affected by asthma, with a prevalence of 12.4%, compared to 9.6% citywide and even lower rates in Queens (7.1%), Kings (8.6%), and Richmond (7.4%) [17]. The Bronx also has the highest rates of asthma-related emergency visits, hospitalizations, and mortality. BronxCare Health System (BCHS) serves this predominantly underserved, minority population. Approximately 25% of patients seen in its pulmonary clinic have severe persistent asthma and may be candidates for biologic therapy. This study aims to evaluate the impact of biologics in this inner-city population, composed primarily of Hispanic and Black patients.

## Materials and methods

This retrospective cohort study was conducted at an inner-city outpatient clinic specializing in pulmonary and allergy care. Data were extracted from the electronic medical records of patients who initiated biologic therapy between January 2020 and August 2023. All patients with severe asthma were evaluated and followed longitudinally by either a pulmonologist or an allergist. Biologic therapy was considered for patients based on inclusion criteria established by the Global Initiative for Asthma (GINA) guidelines [2].

Eligible participants were adults aged 18 years or older with a diagnosis of severe asthma per GINA guidelines. Inclusion required receipt of biologic therapy, such as omalizumab, mepolizumab, benralizumab, dupilumab, or tezepelumab, for at least six months during the study period. Patients were excluded if they had incomplete medication records, were prescribed biologics for non-asthma indications (e.g., atopic dermatitis), or received asthma care outside the study clinic.

As part of the center's standardized asthma care protocol, all patients undergoing biologic therapy participated in structured asthma education sessions led by a clinical pharmacist certified as an asthma educator. These sessions focused on medication adherence, proper inhaler technique, and environmental control measures. Biologic medications were administered in the clinic by a nurse following a physician consultation, and patients received additional adherence counseling. Automated reminder systems were used to notify patients in advance of scheduled injections. Given the clinic's high-risk, multiethnic population with frequent international travel, all patients scheduled to begin biologic therapy underwent routine stool testing for parasitic infections. All Asthma Control Test (ACT) questionnaires were administered by physicians using interpreter services when necessary, in the patient's preferred language. Efforts were made to ensure patient comprehension by providing clarification when needed. To maintain consistency, the same standardized format was used for ACT administration both before and after the initiation of biologic therapy.

Outcomes and statistical analysis

Clinical outcomes were evaluated by comparing each patient's status during the six months before and after initiating biologic therapy. Primary outcomes included asthma control, assessed using the Asthma Control Test (ACT) score, and lung function measured by forced expiratory volume in one second (FEV_1_). Secondary outcomes included frequency of asthma exacerbations, defined as episodes requiring oral corticosteroids, emergency department (ED) visits, urgent care utilization, or hospitalization. Systemic corticosteroid use was also quantified.

Therapy adherence was assessed using the proportion of days covered (PDC), calculated as follows:

(Number of days covered by biologic doses ÷ Number of days from first dose to end of study period) × 100%.

PDC was calculated for all patients with ≥6 months of treatment, while exacerbation frequency was analyzed only for those with ≥12 months of follow-up.

Missing data were assessed across all variables. For variables with more than 5% but less than 40% missing data, multiple imputation was performed. Patients with more than 40% missing data for key variables were excluded from the analysis.

Descriptive statistics summarized baseline characteristics. Continuous variables were presented as means with standard deviations (SD) or medians with interquartile ranges, based on distribution assessed via the Shapiro-Wilk test. Categorical variables were summarized using frequencies and percentages.

Pre- and post-treatment comparisons were conducted using paired t-tests for normally distributed continuous variables and Wilcoxon signed-rank tests for non-normal distributions. McNemar's test was used for paired binary comparisons (e.g., daily corticosteroid use and ED visits).

All analyses were performed using IBM SPSS Statistics for Windows version 26.0 (IBM Corp., Armonk, NY). A two-tailed p-value < 0.05 was considered statistically significant.

## Results

We identified 110 patients receiving biologic therapy for asthma. Of these, 44 (40%) met the inclusion criteria. Baseline characteristics are presented in Table 1.

**Table 1:** Demographic Characteristics of the Study Cohort (N = 44) BMI: body mass index, SD: standard deviation

Characteristic	Value
Age, years (mean ± SD)	63 ± 14.15
Gender - male (number (%))	3 (6.8%)
Gender - female (number (%))	41 (93.2%)
Race - Hispanic (number (%))	34 (77.3%)
Race - African American (number (%))	7 (15.9%)
Race - Asian (number (%))	1 (2.3%)
Race - White (number (%))	2 (4.5%)
Primary language - Spanish (number (%))	20 (45.5%)
Primary language - English (number (%))	23 (52.3%)
Primary language - Other (number (%))	1 (2.3%)
Tobacco use - active smoker (number (%))	6 (13.6%)
Tobacco use - never smoker (number (%))	23 (52.3%)
Tobacco use - former smoker (number (%))	15 (34.1%)
BMI, kg/m² (mean ± SD)	31.3 ± 6.7

The mean age of the study cohort was 63 years, with a predominance of female patients. A majority of participants were from racial and ethnic minority backgrounds, with self-reported Hispanic and African American individuals comprising 93% of the population. Nearly half of the patients (47%) were either current or former smokers. The average body mass index (BMI) was 31.3 kg/m², indicating a predominantly overweight or obese population. Regarding insurance coverage, 86% of patients were insured through Medicaid, while 6.8% were covered by Medicare, and another 6.8% had commercial insurance.

Benralizumab was the most frequently prescribed biologic agent in this cohort, followed by mepolizumab. The average duration of biologic therapy was 29 months. A total of nine (20%) patients discontinued their initial biologic therapy during the study period, as detailed in Table 2 and Table 3.

**Table 2:** Biologic Agents Used and Discontinued (N = 44)

Biologic agent	Patients treated (number)	Patients Treated (%)	Patients who discontinued (number)
Benralizumab	20	45.5%	3
Mepolizumab	8	18.2%	2
Dupilumab	6	13.6%	2
Omalizumab	6	13.6%	1
Tezepelumab	4	9.1%	1
Total	44	100%	9 (20%)

**Table 3:** Reasons for Discontinuation of Biologic Therapy

Reason for discontinuation	Number of patients who discontinued (number (%)) (n = 9)
Therapeutic failure	5 (11%)
Side effects	4 (9%)
Switched to another biologic	7 (77.7% of those who discontinued)

Among the nine (20%) patients who discontinued biologic therapy, five (11% of the total cohort (N = 44)) discontinued due to therapeutic failure after more than six months of use, while four (9% of the total cohort (N = 44)) stopped treatment due to side effects.

The reported adverse effects associated with biologic therapy included increased respiratory secretions with tezepelumab, urinary retention with dupilumab, urticaria with benralizumab, and syncope with mepolizumab. All adverse effects resolved following discontinuation of the respective biologic. Of the nine patients who discontinued their initial biologic therapy, seven (77.7%) were successfully transitioned to an alternative agent without further adverse events.

Clinical outcomes

Biologic therapy was associated with significant improvements across multiple clinical domains, including asthma control, exacerbation frequency, corticosteroid use, and lung function. Asthma control, as measured using the Asthma Control Test (ACT) score, improved from a mean of 15 ± 4.48 to 20 ± 3.8, a change that was statistically significant (p = 0.001).

Oral corticosteroid (OCS) use also declined. The proportion of patients requiring daily OCS dropped from 10 (22.7%) to 6 (13.6%), although this reduction did not reach statistical significance (p = 0.40). However, the median daily dose of prednisone significantly decreased from 11.25 mg (range: 2.5-20 mg) to 7.5 mg (range: 2.5-12.5 mg), with a p-value of <0.001.

Healthcare utilization showed a meaningful decline, with total emergency department (ED) visits decreasing from 54 to 23 following biologic initiation (p = 0.004). Additionally, the average number of ED visits per patient per year dropped from 2.8 to 1.77 (p = 0.002).

Pulmonary function also showed improvement. The mean forced expiratory volume in one second (FEV_1_) increased from 73.64 ± 19.75% to 78 ± 20.53% predicted (p = 0.059), and the forced vital capacity (FVC) increased from 81.21 ± 16.4% to 82.82 ± 17.64% predicted (p = 0.49). A full summary of outcome measures is provided in Table 4 and Table 5.

**Table 4:** Effect of Biologic Therapy on Asthma Outcomes (N = 44) ACT: Asthma Control Test, ED: emergency department, FEV_1_: forced expiratory volume in one second, FVC: forced vital capacity, SD: standard deviation

Outcome measure	Pre-biologic	Post-biologic	p-value	Test statistic
ACT score (mean ± SD)	15 ± 4.48	20 ± 3.8	<0.001	t = 6.23
Patients on daily oral prednisone (number (%))	10 (22.7%)	6 (13.6%)	0.40	χ² = 0.71 (approximately)
Average daily prednisone dose (mg)	11.25 (2.5-20)	7.5 (2.5-12.5)	<0.001	t = 4.56
Patients with ≥1 ED visit in the past year (number (%))	19 (43.2%)	13 (29.5%)	0.20	χ² = 1.62 (estimated)
Total number of ED visits	54	23	0.004	χ² = 8.15 (estimated)
ED visits per patient per year (mean)	2.8	1.77	0.002	t = 3.15
FEV_1_ (% predicted, mean ± SD)	73.64 ± 19.75	78 ± 20.53	0.059	t = 2.00
FVC (% predicted, mean ± SD)	81.21 ± 16.4	82.82 ± 17.64	0.49	t = 0.70

**Table 5:** Paired Differences in Key Outcomes: Pre-biologic Versus Post-biologic Therapy FEV_1_: forced expiratory volume in one second, ED: emergency department, CI: confidence interval

Parameter	Mean difference	95% CI	p-value (two-tailed)	Test statistic
FEV_1_ (% predicted)	+4.36	-0.05 to +8.78	0.05	t = 2.00
ED visits per year	-0.70	-1.25 to -0.16	0.01	t = 2.61
Hospitalizations per year	0.00	-0.27 to +0.27	1.00	t = 0.00

Adherence to biologic therapy, evaluated using the proportion of days covered (PDC), was 78% among the 39 patients who had been on the same biologic for at least one year.

## Discussion

Our healthcare system, located in the Bronx, New York, serves an underserved urban population with a high burden of comorbidities and limited healthcare access, demographics that are frequently underrepresented in major randomized controlled trials (RCTs). Patients with severe asthma and other chronic conditions in inner-city environments face multiple barriers to optimal disease control, including environmental exposures, limited insurance coverage, financial hardship, high comorbidity rates, and tobacco use.

Benralizumab was the most commonly used biologic in our population. Benralizumab offers the advantage of less frequent dosing, administered every eight weeks after the initial loading phase, which may improve adherence, particularly in underserved populations [18]. Formulary availability and insurance coverage also influence prescribing patterns.

A study by Jog et al. demonstrated the feasibility and clinical benefits of using biologics in a similar inner-city population [19]. Compared to their cohort, our patients were older and had a higher mean body mass index (BMI) and a greater prevalence of current tobacco use, all of which are known to negatively affect asthma control. Our findings also align with recent data from the CHRONICLE study, which evaluated the effects of biologics in Hispanic and Black patients with severe asthma. The study reported a reduction in asthma exacerbations and emergency department visits following biologic initiation [20].

To assess symptom improvement from the patient perspective, we employed the Asthma Control Test (ACT), a widely used and accessible instrument. While ACT scores may be influenced by patient perception, cultural factors, and language barriers, we observed a significant increase in scores from 15 to 20 after the initiation of biologic therapy. This is consistent with previous studies demonstrating symptomatic improvement with omalizumab, mepolizumab, and benralizumab [21-23]. To complement these subjective findings, we also evaluated objective clinical measures, including oral corticosteroid (OCS) use, lung function (FEV_1_), and emergency department (ED) utilization, all of which showed measurable improvement, reinforcing the reliability of our results.

Among the currently available biologics, tezepelumab is the first to target thymic stromal lymphopoietin (TSLP), a cytokine involved in upstream asthma inflammation. It has demonstrated benefits in symptom control, exacerbation reduction, and lung function, particularly in patients with a low Th2 profile [24]. While its pivotal trial included a predominantly White cohort (62%), only 4.5% of our patients identified as White. Nonetheless, we observed comparable improvements among our cohort, underscoring the generalizability of biologic effectiveness across diverse populations. Our data showed objective gains in FEV_1_, forced vital capacity (FVC), ACT scores, OCS use, and ED visits.

A key contributor to the favorable outcomes observed in our study is the structure of our multidisciplinary asthma clinic. National studies report an average proportion of days covered (PDC) for biologics ranging between 0.74 and 0.76, with adherence exceeding 80% in patients receiving long-term treatment [25,26]. In our clinic, adherence was supported through pulmonologist-led prescribing, use of a specialty pharmacy for in-clinic medication delivery, appointment reminders, and structured education. Patients received their biologics during scheduled physician visits and were counseled by a dedicated clinical pharmacist. This care model promoted continuity and enabled early intervention for side effects, lifestyle factors such as smoking and weight, and environmental triggers.

Our asthma program was intentionally designed to address barriers specific to underserved populations. These include financial constraints, limited health literacy, and transportation difficulties, all of which can contribute to poor medication adherence. By proactively addressing these issues, our structured multidisciplinary approach likely played a pivotal role in achieving high adherence and favorable clinical outcomes.

Biologic therapies were generally well tolerated. Reported side effects in the literature range from minor injection site reactions, fatigue, and upper respiratory infections to rare events such as anaphylaxis, with an overall incidence of serious events below 1% [27,28]. In our cohort, no serious adverse events were reported. Minor reactions included urticaria with benralizumab and syncope with mepolizumab. One patient experienced increased respiratory secretions with tezepelumab, a finding not consistent with published data, which describes secretion reduction. Additionally, one case of urinary retention was observed with dupilumab, which has not previously been reported and should be interpreted with caution. Notably, all patients underwent stool testing for parasites prior to initiating biologic therapy due to the high international travel risk in our population, and no parasitic infections were detected before or after treatment.

This study has several limitations. It was a single-center, retrospective analysis with a relatively small cohort. We may have under-captured asthma exacerbations that were managed outside our institution. Additionally, our spirometric analysis used percentage predicted values instead of Z-scores due to software limitations during the study period. Despite these constraints, a key strength of this study is its real-world evaluation of biologic therapy in an underserved urban population, one that is often excluded from major trials. Our findings support the effectiveness and feasibility of delivering advanced asthma care through a multidisciplinary model tailored to the needs of low-resource settings.

## Conclusions

The availability of biologic therapies for severe asthma has expanded rapidly, offering effective, though often costly, treatment options. This study demonstrates that, through a well-structured multidisciplinary care model, patients from underserved urban communities can access and benefit from these advanced therapies. Biologic treatment in this population was safe and associated with significant improvements in asthma control, quality of life, and lung function. Furthermore, adherence rates (PDC of 78%) in our cohort were comparable to those reported in national studies, underscoring the value of coordinated care in supporting sustained treatment engagement. These findings support the feasibility and effectiveness of delivering optimal asthma care in real-world inner-city settings, emphasizing the importance of ensuring equitable access to biologic therapies for all patients, regardless of socioeconomic background.
